# The Impact of Boundary Spanning Scholarly Publications and Patents

**DOI:** 10.1371/journal.pone.0006547

**Published:** 2009-08-18

**Authors:** Xiaolin Shi, Lada A. Adamic, Belle L. Tseng, Gavin S. Clarkson

**Affiliations:** 1 Department of EECS, University of Michigan, Ann Arbor, Michigan, United States of America; 2 School of Information, University of Michigan, Ann Arbor, Michigan, United States of America; 3 Yahoo Inc., Sunnyvale, California, United States of America; 4 University of Houston Law Center, Houston, Texas, United States of America; Aarhus University, Denmark

## Abstract

**Background:**

Human knowledge and innovation are recorded in two media: scholarly publication and patents. These records not only document a new scientific insight or new method developed, but they also carefully cite prior work upon which the innovation is built.

**Methodology:**

We quantify the impact of information flow across fields using two large citation dataset: one spanning over a century of scholarly work in the natural sciences, social sciences and humanities, and second spanning a quarter century of United States patents.

**Conclusions:**

We find that a publication's citing across disciplines is tied to its subsequent impact. In the case of patents and natural science publications, those that are cited at least once are cited slightly more when they draw on research outside of their area. In contrast, in the social sciences, citing within one's own field tends to be positively correlated with impact.

## Introduction

Applying bibliometrics to citation networks to study the impact of fields, individuals, and particular papers has been the purview of the field of scientometrics [1]. It was already in the 1960s that de Solla Price first developed models to explain the heavy tailed distribution in the citations an individual paper receives [2]. Recently, the availability of large scale citation data and computational power has enabled the visualization and quantification of the amount of information flow between different areas in science [3], [4], in effect mapping human scientific knowledge. These visual maps leave open the question, however, of the size, speed and impact of information flows across community boundaries. Prior work has shown these flows to be relatively insignificant; omitting information flow between communities when one models citation networks still provides realistic citation distributions and clustering coefficients [5], [6]. Not only are information flows across scholarly communities infrequent, they are also delayed: on average more time elapses between the citing and cited articles for citations across disciplines than ones within a discipline [7].

Through their specialized organizations, activities, and publication venues, disciplines facilitate the frequent and timely dissemination of information. Within-discipline communication allows individuals to be exposed to research that is closest and most relevant to their own. Yet, there is a belief, reflected in many cross-disciplinary initiatives, both at the university and government levels, that knowledge flows between disciplines are not only beneficial, but are more likely to lead to innovative and groundbreaking research.

There is some evidence that interdisciplinary collaborations do lead to higher impact work. A study of scholarly articles in the UK found that papers whose coauthors are in different departments at the same university receive more citations than those authored in a single department, and those authored by individuals across different universities yield even more citations on average [8]. Multi-university collaborations that include a top tier-university were found to produce the highest impact papers [9]. Similarly, in the area of nanotechnology authors who have a diverse set of collaborators tend to write articles that have higher impact [10]. Interdisciplinarity aside, new collaborations between experienced authors are more likely to result in a publication in a high impact journal than new collaborations with an unseasoned author or repeat collaborations between the same two authors [11]. The argument is that merging ideas and expertise in a novel way will produce higher impact work. It has also been demonstrated that scholarly work in a range of fields and patents generated by larger teams of coauthors tends to have greater impact over time [12]. However, in the above studies examining author collaborations, there may be confounding factors. For example, successful authors may consequently have more opportunity to collaborate across departments and universities due to higher motivation or visibility.

In this paper we aim to measure the impact of information flows from one field to another more directly by tracing citations. Citations often, but not always, indicate that knowledge from one publication is being incorporated in another. Authors of the citing paper have found the other paper relevant, and more importantly, have usually, though not always [13], read it. Sometimes authors cite others where social norm or strategic positioning may encourage citation. Such behavior, if successful, would tend to reward citations within the same community or discipline, where one is targeting a publication. In the context of patents, inventors cite inventions that their own patent depends on or may be a substitute for.

We use as an indication of quality and impact of the work the number of citations a paper or patent receives normalized by the average number of citations received by all papers or patents in the same area and year [14]. This measure allows us to make a fair comparison between articles that may not have finished accumulating citations due to their recency, and to account for differences in size and publication cycle for different disciplines [15]. We take each individual citation as evidence of information flow, whether within a field or between fields.

The question we ask is simple: given the proximity in subject area between a citing publication (paper or patent) and cited publication, what is the impact of the citing publication? If cross-disciplinary information flows result in greater impact, one would see a negative correlation between proximity and impact. On the other hand, if it is within-discipline contributions that are most easily recognized and rewarded, one would observe a positive correlation.

## Methods

Our analysis uses two large data sets. The first, provided by JSTOR (Journal Store), has 1.98 million research articles in 1108 journals, classified into 47 disciplines, roughly corresponding to 3 sets: arts & humanities, social sciences, and the natural sciences. Of those, there are 655,213 research articles citing 722,152 other articles within the dataset, for a total of 5,598,657 citations. These citations, limited to the cases where both the citing and cited articles are in the dataset, are a subset of the 23,451,235 citations made by the articles in total. Similarly, when measuring impact, we only count the number of citations from within the dataset. Although this could skew the observed raw citation counts toward disciplines that are better represented within the dataset, the normalization by discipline mitigates such biases. The patent data set contains all 5,529,055 patents filed between 1976 and 2006, and 2348 different categories with at least 1000 patents. There are 3,643,520 patents citing 2,382,334 others, for a total of 44,556,087 citations. The citation impact information is complete, since the dataset contains all subsequent patents.

Our analysis proceeds by examining each individual citation, the proximity of the disciplines of the citing and cited article for that citation, and the impact of the citing article. Intuitively, any individual citation will at most have a very weak impact on the success of a citing paper. It will only be one of possibly dozens of references made in an article or patent. Other factors, such as the publication venue and the reputation of the authors, are more likely to contribute to the impact of the article than any individual citation the authors include. We nevertheless see a significant relationship between the interdisciplinarity of citations and the impact of the publication.

We assign disciplines to an article according to the JSTOR classification of the journal; approximately half of the journals are assigned to just one discipline, while the rest have multiple assigned disciplines. Each patent is assigned by a USPTO patent examiner to one or more categories according to the USPTO classification system. We quantify the proximity between disciplines by comparing the number of citations between any pair of disciplines relative to the rate of citation we would expect if the volume of inbound and outbound citations were the same, but the citations were allocated at random. If a citing or cited journal is classified into more than one discipline, a fractional citation is attributed to each discipline. We let 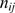 be the actual number of citations from 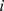 to 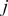, 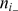 be the number of outbound citations from discipline 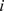, 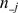 be the number of inbound citations to discipline 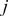, and 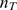 be the total number of citations. Then the expected number of citations, assuming indifference to one's own field and others, from field 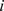 to field 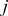 is 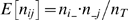. We define the directed proximity as a Z-score that tells us how many standard deviations above or below expected 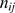 is:
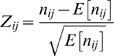



Here we have used the observation that 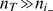 and 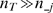, and approximated the standard deviation by 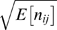.

**Figure 1:**
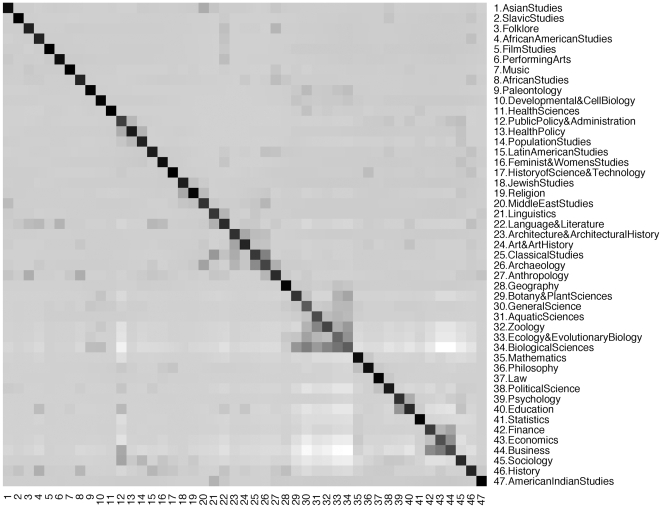
Information flow matrix for journals in the JSTOR database. The direction of information flow is from the column discipline to the row discipline, with 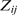, the Z-score, corresponding to the 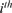 row and 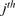 column. Each entry is shaded according to a normalized Z-score representing whether the number of citations between disciplines is higher or lower than expected at random. Darker shading represents higher Z-scores. The diagonal represents citations within the same discipline.

A high proximity between areas 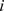 and 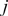 indicates a strong tendency for papers or patents in area 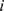 to cite publications in area 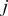. Figure 1 shows an information flow matrix of proximities by pairs of disciplines in JSTOR. Unsurprisingly, a discipline is most likely to cite itself. But one can also observe a tendency of the natural sciences to cite one another, while the natural and social sciences have fewer cross-citations. Furthermore, although the proximity from area 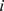 to area 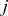 is highly correlated with proximity from 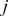 to 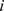 (with a Pearson correlation of 0.968), the measure also captures any underlying asymmetry in citation patterns. Typically the more applied fields cite the more basic ones slightly more often. Note that our measure is an aggregate over the entire lifetime of the journals included, and that previous time resolved measurements of information flow in chemisty-related fields have detected changes in flow as fields evolve [16].

In our aggregate sample, Finance cites Economics more often than Economics cites Finance. Statistics is more often cited by other fields than it cites them, with the exception of Mathematics. The areas of Zoology and Botany and Plant Sciences cite the Biological Sciences more often than the Biological Sciences cite them. These asymmetries also reflect how unusual a citation is. A Biology paper citing a Statistics paper would be unusual, and might indicate the incorporation of a non-standard method. A Statistics paper citing a Biology paper would be even slightly more unusual, and might signal a motivation for the development of a novel method.

**Figure 2:**
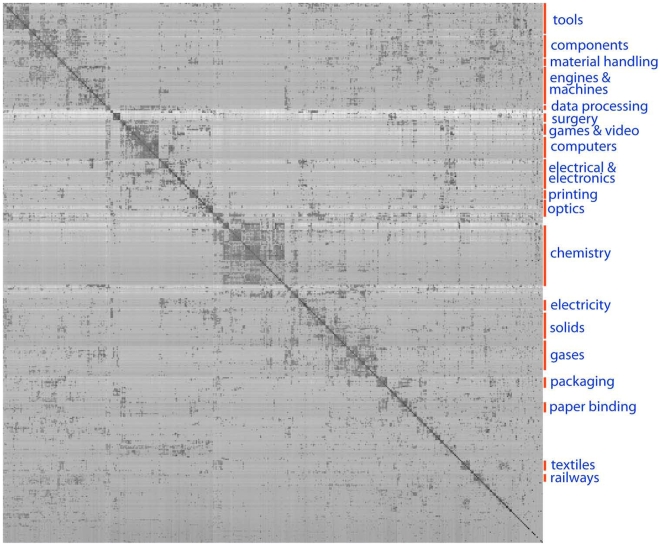
Information flow matrix for patents, with several related areas labeled.

Figure 2 shows the information flow matrix for patents. For purposes of visualization, we have aggregated all citations according to 468 top level classifications (e.g. 029 corresponds to “metal working” while 901 corresponds to “robots”). We similarly observe a tendency of patents within the same subject classification to cite one another (patents are typically classified into several classes). Once more the proximity measure reveals asymmetries in information flow. For example, patents in category 623 “Prosthesis”, which includes pacemakers for the heart, cite category 433 “Horology” more often than vice versa. Category 277, having to do with seals for a “joint or juncture” is more often cited by the categories corresponding to pumps and wells than it cites them. In general, those categories representing basic components and methods have a net surplus of citations, and include e.g. machine elements of mechanisms, gas separation, adhesives, stock material, and cryptography, among others. However, sometimes a category corresponding to a complex apparatus or process, such as 358 “Facsimile and static presentation processing” also has a net surplus of citations. This may occur when an invention matures and precedes other related inventions. The facsimile category is cited many times by other categories that developed later: television, computers, computer graphics, and interactive video.

In order to test the sensitivity of our results to our particular choice of proximity measure, in addition to the simple ratio of observed to expected citations, we also use the Jaccard coefficient for the sets of authors publishing in two areas. We select the latter measure because it is very different from citation-based metrics, while still capturing proximity. An author could much more easily cite an unrelated area than they could directly contribute to it by publishing in that area's journals. In further contrast to the Z-score metric, the Jaccard coefficient is an undirected measure. Yet we still find our results, reported in Text S1, to be quantitatively and qualitatively consistent.

## Results

For every citing relationship, we measure the Spearman correlation between citation proximity and the impact of the citing publication. Citation proximity is simply 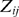, where 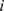 is the area of the citing publication, and 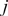 is the area of the cited publication. If a paper or patent belongs to more than one area, the proximities are averaged. We sought to measure impact consistently across the diverse areas represented by our data sets. To that end, we measured impact (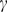) as the the number of citations received by the citing publication, normalized by dividing by the average citation count of a publication in the same year and area(s).

We find that for the entire patent data set the correlation is positive with 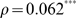 (***, **, and * denote significance at the 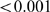, 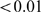 and 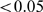 levels respectively). The corresponding correlation for natural science papers in JSTOR is slightly negative with 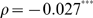. However, one can also focus on publications with at least a given level of success. First, we omit the 40.03% of patents and 34.46% of natural science papers that were never cited within our datasets. After removing these zero-impact publications, the tendency of within-community citations to be rewarded is more significantly negative for both the natural science papers and patents: for patents, this correlation is 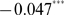 and for natural science papers, the correlation is 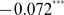. This result suggests that a publication citing within its discipline is more difficult to ignore altogether. However, given that a natural science publication or patent attracts at least some attention, there is a slight tendency for those that cite outside of their area to have higher impact.

**Figure 3:**
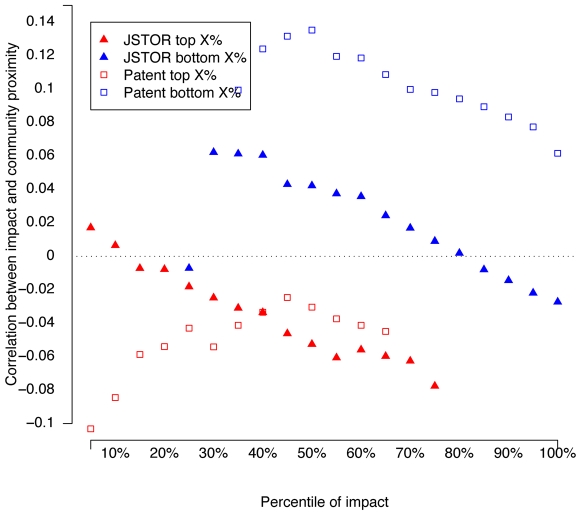
Correlations between proximity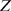 and impact 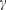, partitioned by percentile of impact. For example, at the 20% percentile, we show 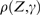 for the bottom 20% of publications by their impact 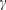, and for the top 20% by 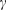. No correlations are shown for the bottom 10–20% of publications because they received no citations.

To demonstrate that the result is not dependent simply on removing papers with no citations, we also slice the data according to percentile of impact, e.g. taking the bottom 30% and top 30%, and calculating correlations between citation proximity and impact separately for the top and bottom group. As Figure 3 shows, we consistently observe a negative correlation between citation proximity and impact for the higher impact group.

**Figure 4:**
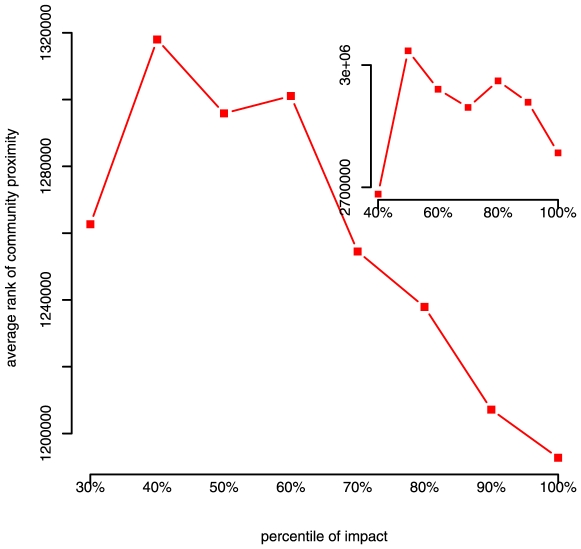
Average community proximity of citations by impact of citing article in JSTOR. The inset shows the average trend for patents.

Figure 4 helps to explain why removing zero and low impact publications leaves a negative correlation between citation proximity and impact. By plotting mean proximity as a function of impact, we observe that both very low and very high impact papers tend on average to cite outside of their area more often. Since very low impact publications include many publications that cited outside of their discipline but failed to attract notice, we are left with the portion of cited publications where citing outside of ones discipline is positively correlated with impact. These results suggest that citing outside one's discipline is a gamble. While risking not being cited at all, publications that incorporate work from other disciplines tend to make more significant contributions.

Interestingly, the correlation between the interdisciplinarity of citations and the impact of a publication in the social sciences and humanities remains positive to neutral regardless of whether one includes or excludes zero citation publications. In the social sciences the correlation is 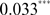 when zero impact publications are included, and 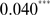 if they are excluded. The correlation for the entire set of humanity papers is 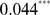, and 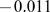 (not sig.) after removing papers with zero impact. That citing outside of one's discipline has different implications depending on whether one is a natural or social scientist is an interesting observation for further study.

In the above analysis, the correlation values are obtained individually by correlating the citation proximity and the impact of the citing publication for each citation pair. One can, however, also consider the average community proximity between a given publication and all of the publications it cites. Note that these averages are not always representative because many cited publications fall outside of our datasets. Nevertheless, the correlation is 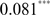 for the entire set of patents, and 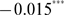 for the set of patents having non-zero impact. For JSTOR, the correlations are 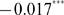 and 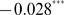 respectively for the set of natural science publications. These correlations are weaker, though consistent with the correlations obtained for individual citation pairs.

In order to interpret this result we should consider two scenarios for why an inter-community edge would appear. The first is that an author publishes in a venue outside their usual area, but cites work from their home area. It may be expected that their impact in the venue is diminished, possibly because the publication is of peripheral interest, or the Matthew effect [17] is absent, since the author has not already built up a reputation at that venue, and her work is less likely to be noticed. A second possibility is that an author who usually publishes in a given venue draws upon another field in their work, sometimes by co-authoring directly with someone from another discipline [10]. One may expect such work to have potentially higher impact, since it is bringing in knowledge that could have greater novelty. Unlike journal publications where one may expect that impact will depend on both a suitably chosen venue and the innovativeness of the work, for patents there is only a single venue, the US patent office. Nevertheless, a patent's classification, determined by the patent office, affects its likelihood of being found by examiners and inventors searching the patent database.

**Figure 5:**
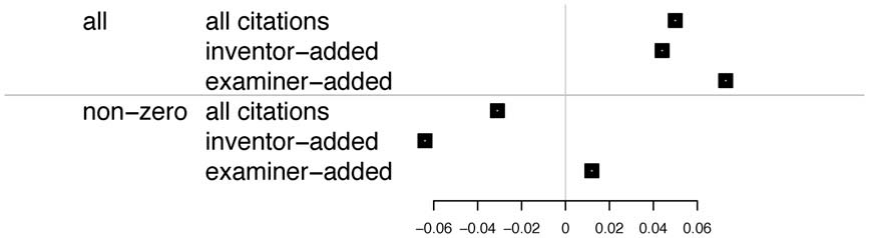
Correlations between citation proximity and impact, for patents published between 2000 and 2006, separated by whether the citation was added by an inventor or patent examiner.

Another way in which patents differ from journal articles is in the origin of the citations. As many as two thirds of all patent citations are added not by the inventors, but by the patent examiners, and it is therefore unlikely that such citations represent true knowledge flows [18]. Fortunately, since 2000, examiner-added citations are delineated from inventor-added ones. Already in the choice of patents to cite we find that examiners are more specialized in their citations than inventors; the average proximity for citations added by examiners is 213.471, compared to 155.572 for those added by inventors. Figure 5 shows that, unlike inventor added citations, examiner-added citations show a neutral to positive correlation for citing patents in proximate categories. This suggests that patent examiners may not only be biasing citations to fall within categories, but when they do, the patent is more likely to receive citations.

Finally, we combine proximity with other variables which may influence the impact of the publication or patent. We include network properties of the citing and cited publications in the citation graph as well as the time of publication for both. We exclude variables such as publication venue and author since these themselves may be correlated with the likelihood of cross-disciplinary information flows. Table 1 gives the coefficients of the variables of the regression models. The dependent variables in these models are the impact of the citing paper of each citation pair after applying a Box-Cox transformation with an appropriate 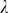, i.e: 
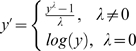



**Table 1:** Citing behavior and subsequent citations earned.

variable	US Patents		Natural science papers in JSTOR	
	all _(λ = 0.35)_>0 cites _(λ = 0)_		all _(λ = 0)_>0 cites _(λ = −0.069)_	
log(#cited_citing_+1)	1.816e-01^***^	1.543e-01^***^	7.605e-01^***^	3.577e-01^***^
log(#citations_cited_+1)	1.470e-01^***^	1.047e-01^***^	2.635e-01^***^	9.971e-02^***^
citing year	−1.096e-02^***^	5.195e-05^***^	−1.019e-02^***^	−7.828e-03^***^
year difference	−1.697e-02^***^	−1.092e-02^***^	−1.962e-02^***^	−7.209e-03^***^
**proximity**	−5.873e-10^***^	−1.586e-08^***^	−1.743e-09^***^	−1.735e-08^***^
*R* ^2^	0.0672	0.0534	0.1570	0.1018
citation pairs	2,841,279	2,683,726	2,110,965	1,729,298

*p*<0.05(^*^), *p*<0.01 (^**^), *p*<0.001 (^***^).

Because of the extreme ly skewed distribution of the values of community proximity, we use their ranks instead of their normalized Z-score values. From Table 1, we see, consistent with results in Figure 3, that even controlling for other variables, cross-disciplinary citations correlate with higher impact for non-zero impact publications.

Furthermore, citing well-cited publications corresponds to receiving more citations, as does citing more recent publications. This is interesting in light of the recent finding that electronic access tends to make it easier to cite more recent and more influential papers [19]. Finally, citing many other publications positively correlates with receiving more citations. One might speculate that a publication that carefully acknowledges and builds upon a substantial body of previous work will itself be relevant to a wider range of future work.

**Figure 6:**
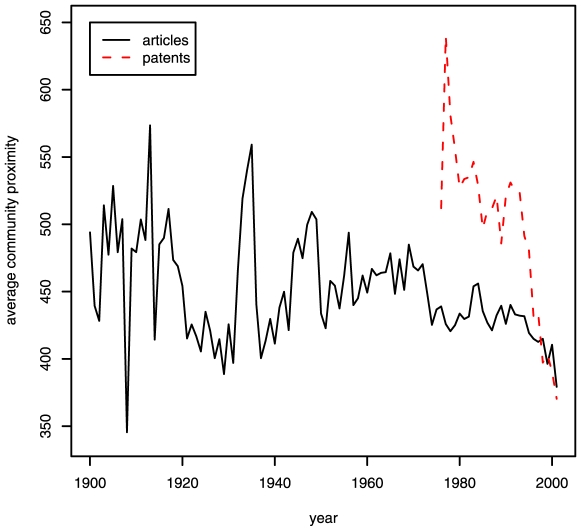
Average community proximity between communities over time.

Given the higher impact of information flows spanning disciplines, an important question one might ask is whether interdisciplinary citations have increased in recent years. Figure 6 shows the evolution of average community proximity over time for patents and for papers in JSTOR. We observe that the frequency of citations crossing communities among scholarly work has remained approximately constant over the past 100 years. For patents, we observe a mild increase in interdisciplinary citations from 1975 to 1990 and a sharper increase thereafter. This indicates that even though the amount of knowledge has been accumulating within each area, patent inventors and examiners are increasingly identifying and building upon relevant inventions in other areas. Note that our measures of proximity are based on the cumulative citation counts for the entire period of the datasets, which does not take into account variations in proximity between pairs of disciplines over time. Because of this, some pioneering papers that bring together disciplines before such cross-disciplinary research becomes common, may not be recognized in our analysis. On the other hand, the average author Jaccard coefficient 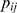 for citations among patents and papers in JSTOR, shown in Figure S1, is decreasing to constant, as was the case for the community proximity measure 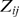 shown in Figure 6.

In summary, we quantified through a bibliometric analysis the effect of interdisciplinary information flows. We found that among patent inventions and natural science papers receiving one or more citations, those who cite across disciplines tend to garner more citations, indicating that cross-fertilization of ideas does often lead to significant impact.

## Acknowledgments

We thank IBM for providing the patent data and JSTOR for providing the article citation data. We would also like to thank Michael McQuaid for insightful discussions.

## Supporting Information

Figure S1 Average $p_{ij}$ between communities over time.
(0.24 MB EPS)

Text S1 (0.03 MB PDF)
