# Supplementary material for: The Impact of Boundary Spanning Scholarly Publications and Patents
Source: PLoS One. 2009 Aug 18;4(8):e6547. doi: 10.1371/journal.pone.0006547 (PMC2722725; doi:10.1371/journal.pone.0006547)
Supplement: Text S1 — (0.03 MB PDF) [file pone.0006547.s002.pdf]

Text S1

Alternate definitions of proximity between communities In addition to defining proximity in terms of citation frequency between areas/categories, one can also define it in terms of the author Jaccard coefficient p_{ij} that measures the ratio of the number of authors who publish in both areas to the number of authors who publish in either. Using the Jaccard coefficient has the feature of being 1 for all within community citations, and 0 for two areas that share no authors. In contrast, the community proximity measure has different weights for within-community citations because the Z-score measures how many more within-community citations than expected one observes, which varies by area. Therefore the Jaccard coefficient is able to treat all within-community citations equally.

We find generally good agreement between the two measures when correlated against impact. For patents overall, the correlation drops to slightly negative using author overlap ($\rho = -0.008^{***}$), but is again significantly negative once the zero impact patents are removed ($\rho = -0.034^{***}$). Similarly for natural science articles in JSTOR, the non-zero impact articles have more significantly negative correlation ($\rho = -0.080^{***}$) compared with the overall correlation ($\rho = -0.037^{***}$). Once again, we have the result that inventions and natural science publications citing outside of their area tend to have slightly higher impact. For the humanities and social sciences, the correlations remain significantly positive both before and after excluding zero impact publications.
